# Perinatal Distress in Fathers: Toward a Gender-Based Screening of Paternal Perinatal Depressive and Affective Disorders

**DOI:** 10.3389/fpsyg.2020.01892

**Published:** 2020-08-18

**Authors:** Franco Baldoni, Michele Giannotti

**Affiliations:** ^1^Department of Psychology, University of Bologna, Bologna, Italy; ^2^Department of Psychology and Cognitive Sciences, University of Trento, Trento, Italy

**Keywords:** fathers, perinatal paternal depression, screening, affective disorders, depressive symptoms

## Introduction

In Western society, with the gradual transition from the patriarchal family to the contemporary nuclear one (both parents work and contacts with the extended family are limited), fatherhood has been increasingly linked to more expectations and responsibilities in childcare and family life (Quilici, 2010; Biehle and Mickelson, 2012; Crespi and Ruspini, 2015). At biological level, neural plasticity and hormonal changes that occur in men may also increase the risk of psychological distress during the transition to parenthood (Poromaa et al., 2017; Baldoni, 2020). Research has clearly demonstrated that during the perinatal period the emotional states of mothers and fathers influence each other showing a significant correlation between paternal and maternal perinatal depressive disorders (Baldoni and Ceccarelli, 2010; Paulson and Bazemore, 2010; Musser et al., 2013).

Thus, in the last decades there has been an increased interest in men's perinatal mental health (Baldoni, 2010; Garfield, 2015; Gutierrez-Galve et al., 2015; Field, 2018). In this scenario, Paternal Perinatal Depression (PPND) is considered a specific condition that affects many fathers between pregnancy and the first year after childbirth. PPND is associated with maternal depression (Baldoni et al., 2009; Paulson et al., 2016) and adverse outcomes in children and adolescents, including externalizing and internalizing symptoms (Ramchandani and Psychogiou, 2009; Baldoni, 2016; Sweeney and MacBeth, 2016). Specifically, a longitudinal study on 12,884 fathers has confirmed the influence of PPND on the psychophysical development of children evaluated from birth to 7 years of life (Ramchandani et al., 2005, 2008), with an increase, more significant in males, in emotional and behavioral control problems at 21 and 42 months and childhood psychiatric disorders and oppositional behaviors at 7 years.

Other studies (Baldoni et al., 2009, 2011) conducted with the CARE-Index (Crittenden, 1979–2007) documented the influence of depression and poor paternal sensitivity on the psychomotor development of infants (assessed with the Bailey scales).

Two recent metanalyses showed a PPND prevalence in the word ranging from 10.4% (Paulson and Bazemore, 2010) to 8.4% (Cameron et al., 2016) and longitudinal studies found that pregnancy is the most sensitive period for the onset of symptoms in both men and women (Madsen and Juhl, 2007; Figueiredo and Conde, 2011). Therefore, the term *Paternal Perinatal Depression* (PPND) is gradually replacing *Paternal Post-partum/Post-natal Depression (PPD)*, to consider and identify the possible onset of depressive symptoms in fathers since the prenatal period (Baldoni, 2010; Cameron et al., 2016; Bruno et al., 2020). Although these terms are commonly used in research, these diagnoses are not even mentioned in the current DSM-5. The manual only specifies the criteria for a major depressive episode “with peripartum onset” referring to the mother only, which is defined as the most recent episode occurring during pregnancy as well as in the 4 weeks following delivery.

Anyhow, fathers are not usually the focus of the prevention and screening of perinatal affective disorders, and PPND remains underestimated and undertreated compared to maternal depression. A possible explanation is that men tend to show a less clear clinical picture than women do and thus the use of screening questionnaires developed for mothers may be not appropriate. Given that perinatal depression risks and psychological responses differ significantly based on gender (Habib, 2012), it would be useful to rethink perinatal psychological disorders considering the wide array of paternal affective symptoms and the limitations of current tools developed to assess maternal depression. Hence, the aim of this opinion article is to emphasize the need to consider male-specific responses to perinatal distress following an integrative and gender-based perspective. Secondly, we have commented on the limits of research on PPND based on current self-reported measurements.

## Paternal Perinatal Depression: A Complex Clinical Picture

Expression of PPND differ from that of *Maternal Perinatal Depression* (MPND) in terms of intensity and clinical picture, even if time of onset, and duration can be similar.

Owing to psychosocial influences, males tend to display emotional suffering through externalizing and behavioral symptoms rather than typically depressive-like responses (Baldoni, 2016; Seidler et al., 2016). In fact, compared to MPND, PPND may occur along with other disorders whose symptoms may overlap or mask it (Abramowitz et al., 2001; Goodman, 2004; Baldoni, 2010, 2016; Martin et al., 2013; Madsen, 2019; Bruno et al., 2020). The most common are anxious disorders, abnormal illness behavior, behavioral acting outs, and addictions. Given the frequent comorbidities, we proposed (Baldoni, 2016) to replace the term PPND with *Paternal Perinatal Affective Disorder* (PPAD) using a more comprehensive definition to encompass the broad range of depressive equivalents associated with male psychological perinatal distress. Accordingly, considering these different areas, an appropriate assessment of fathers should start from early prenatal period (Baldoni, 2010, 2016).

### Depressive Symptoms

Perinatal depressive symptomatology in fathers is generally milder and less defined than in mothers. It may consist in vague experience of depressed mood, restlessness, irritability, loss of interest, attention difficulties, reduced work output, social isolation, withdrawal from close relationship, loss of or increased appetite, loss of sexual desire and insomnia. Clinicians can often underestimate most of these symptoms, excepting for depressed mood, considering them as manifestations that normally occur during the perinatal period.

### Anxious Symptoms

Starting from the prenatal period a relation between depressive and anxious symptomatology has been observed in fathers (Fletcher et al., 2006; Wee et al., 2015; O'Brien et al., 2017; Chen et al., 2019). Anxiety disorder (GAD, panic attacks, PTSD) may be even more frequent than typical depressive symptoms in men (Wynter et al., 2013). Recent findings showed a prevalence ranging from 4.1 to 16% before childbirth and a stable course across the perinatal period (Leach et al., 2016). In an Australian study (Matthey et al., 2003), among 196 fathers, 9.7% met DSM-IV criteria for anxiety disorder, and only 1% met criteria of depression. In this regard, the definition *Perinatal Mood Disorder* has been proposed to consider both anxious and depressive symptoms as crucial targets of the screening practice.

### Abnormal Illness Behavior

Illness behavior refers to the way people react to their own body functioning in terms of health or illness. This aspect is crucial given that the expression of physical distress has been associated with PPND (Danielsson and Johansson, 2005) and research highlighted the presence of hypochondria, somatization, or functional medical syndromes in partners of depressed mothers (Baldoni et al., 2009; Martin et al., 2013). These disorders may overlap with a *Couvade syndrome* (Trethowan and Conlon, 1965; Baldoni, 2016) e.g., the manifestation in the father of somatic complains, female behaviors, and pregnancy concerns that rarely take on significant psychopathological value.

### Anger Attacks and Behavioral Acting Outs

The scientific community has recognized the need to assess behavioral *acting outs* and *loss of impulse-control* in the screening of PPND (Martin et al., 2013; Madsen, 2019). Anger attacks, violence, compulsive physical, or sexual activities, extra-marital relations, fugues from home or at work may accompany or mask a depressive symptomatology (Baldoni, 2016). Furthermore, it is necessary to consider the higher suicidal risk in depressed males (Innamorati et al., 2011), which could often be manifested through suicidal equivalents (e.g., unnecessarily exposure to serious danger, practicing harmful, or risk-taking activities).

### Addictions

Males during perinatal period tend to report higher rates of substance use (alcohol, smoking, or drugs) or other addictions (e.g., gambling, compulsive use of computer, smartphone, or internet) (Baldoni, 2016; Madsen, 2019). This response generally constitutes an attempt to control dysregulated mental states and somatic functions that accompany them. Distressed fathers can use these behaviors to calm down, get excited, overcome boredom, or distraction (Baldoni, 2016).

## Self-Reported Measures for the Screening of At-Risk Fathers

Although gender-related differences in the expression of perinatal affective disorders have been sufficiently recognized (Baldoni, 2010; Martin et al., 2013), little attention has been paid to the assessment of these problems in males (Psouni et al., 2017). Usually research and screening are based almost exclusively on self-report tools that only consider symptoms associated with maternal perinatal depression. Currently, only the *Gotland Male Depression Scale* (GMDS; Zierau et al., 2002), the *Masculine Depression Scale* (Magovcevic and Addis, 2008), and the *Male Depression Risk Scale* (MDRS-22; Rice et al., 2013) are the specific tools to assess male symptomatology, but they have not been developed for the perinatal period. The well-known *Edinburgh Post-natal Depression Scale* (Cox et al., 1987), developed for mothers, has been validated in fathers also (Matthey et al., 2001; Edmondson et al., 2010; Lai et al., 2010; Loscalzo et al., 2015) using lower cut-off scores to detect major depression and anxiety disorders in the latter. However, there is still no agreement on the optimal cut-off scores for depression and anxiety, which vary across studies. Although an elevated level of sensitivity was found with a cut-off of 12, it has been suggested that EPDS may not be appropriate for mild-minor depression and anxiety disorder (Massoudi et al., 2013). Moreover, the well-established two-factor structure of the EPDS in mothers (depression and anxiety) was not replicated in any of the studies on fathers (Matthey, 2008; Massoudi et al., 2013; Loscalzo et al., 2015). Given the different factorial structure, previous research suggested not using the EPDS to screen for anxiety disorder in fathers (Matthey, 2008). According to a recent metanalysis (Cameron et al., 2016), studies based on EPDS used multiple cut-offs to determine depression generating little comparable results and the authors pointed out the need to standardize cut-off scores to produce a more consistent literature. In addition, other studies used two different questionnaires to assess perinatal depression in fathers providing contradictory results. In particular, in a Japanese study (Nishimura and Ohashi, 2010) the CES-D (*Center for Epidemiological Study Depression Scale*) (cut-off ≥ 16) and the EPDS (cut-off ≥ 9), revealed different findings (7.5% of fathers exceeding the CES-D cutoff, whereas 11.6% the EPDS cut-off). In a Danish study (Madsen and Juhl, 2007) it was estimated that 5% of men assessed by EPDS reported post-natal depression, but using the GMDS the average dropped to 3.4%. Notably, 20.6% of the at-risk fathers in this sample exceed the cut-off value only on the GMDS. Similarly, Carlberg et al. (2018) found that EPDS and GMDS were associated with different risk factors and prevalence of PPND, suggesting that a significant number of at-risk fathers would not be detected by one instrument alone. It is plausible that the two questionnaires cover different aspects of paternal perinatal distress. In fact, a recent study (Psouni et al., 2017) revealed that a combination of the two measures showed higher sensitivity than EPDS alone. It is also interesting to note that a specific subgroup of fathers only exhibits externalizing depressive equivalents without conventional symptoms.

Recently, a team of researchers developed the *Perinatal Assessment of Paternal Affectivity* (PAPA) (Baldoni et al., 2016a,b, 2018) a new self-report questionnaire for the screening of affective symptoms in fathers. This tool is based on recent research on perinatal affective disorders and assesses different dimensions of paternal affective suffering: anxiety, depression, irritability/anger, couple and relational difficulties, somatic complaints, risky behaviors, and addictions (smoking, alcohol, drugs, gambling, internet abuse, physical or sexual compulsive, and risky behavior) considering also some ethnic and socio-cultural factors.

## Conclusion

Contemporary research has highlighted the need to assess perinatal distress using gender-specific tools for mothers and fathers (Walsh et al., 2020). It is essential to develop new instruments to evaluate a broad range of depressive equivalents increasing the sensitivity and specificity of the screening (Matthey et al., 2001; Baldoni, 2016; Baldoni and Giannotti, 2017; Psouni et al., 2017; Madsen, 2019). Currently, a diagnosis of *Paternal Perinatal Affective Disorder* (PPAD) may reflect a more integrated and inclusive perspective to evaluate men's mental health during the perinatal period. This approach may help in reducing sex disparities and mother-centered bias in the screening practice of the perinatal affective disorders. An appropriate screening of at-risk fathers should constitute an essential prerequisite for perinatal health services, given the impact of psychological distress on maternal health, family adaptation, and child development.

## Author Contributions

All authors listed have made a substantial, direct and intellectual contribution to the work, and approved it for publication.

## Conflict of Interest

The authors declare that the research was conducted in the absence of any commercial or financial relationships that could be construed as a potential conflict of interest.

## References

[B1] Abramowitz J. Moore K. Carmin C. Wiegartz P. S. Purdon C. (2001). Acute onset of obsessive-compulsive disorder in males following childbirth. Psychosomatics 42, 429–431. 10.1176/appi.psy.42.5.429 11739911

[B2] Baldoni F. (2010). Attachment, danger and role of the father in family life span. Transilv. J. Psychol. 4, 375–402.

[B3] Baldoni F. (2016). “I disturbi affettivi perinatali nei padri,” in Manuale di Psicologia Perinatale, eds P. Grossu and A. Bramante (Milano: Erickson), 443–485.

[B4] Baldoni F. (2020). Essere padre di un bambino nato pretermine. Psichiatria e Psicoterapia 39, 225–238.

[B5] Baldoni F. Baldaro B. Benassi M. (2009). Affective disorders and illness behaviour in perinatal period: correlations between fathers and mothers. Child Dev. Disabil. 36, 25–44.

[B6] Baldoni F. Ceccarelli L. (2010). La depressione perinatale paterna. Una rassegna della ricerca clinica ed empirica. Infanzia e Adolescenza 9, 79–92.

[B7] Baldoni F. Facondini E. Romeo N. Minghetti M. Cena L. Landini A. (2011). “Attachment forerunners, dyadic sensitivity and development of the child in families with a preterm born baby,” in Proceedings 6th European AEPEA Congress on Psychopathology in Childhood and Adolescence (Bologna 5-7 Maggio), 422–430.

[B8] Baldoni F. Giannotti M. (2017). “I disturbi affettivi perinatali paterni: valutazione, prevenzione e trattamento,” in Psicologia Clinica Perinatale. Neuroscienze e Psicoanalisi, eds A. Imbasciati and L. Cena (Milano: Franco Angeli), 218–239.

[B9] Baldoni F. Matthey S. Agostini F. Schimmenti A. Caretti V. (2016a). Perinatal Assessment of Paternal Affectivity (PAPA): preliminary report on a new screening tool. World Association for Infant Mental Health (WAIMH) Congress (Prague, May 29–June 2). Infant Mental Health J. 37(Suppl. 1), 132–133.

[B10] Baldoni F. Matthey S. Agostini F. Schimmenti A. Caretti V. (2016b). Perinatal Assessment of Paternal Affectivity (PAPA) Manual (version 3.1). Bologna: Department of Psychology, University of Bologna.

[B11] Baldoni F. Matthey S. Agostini F. Schimmenti A. Caretti V. (2018). Perinatal Assessment of Paternal Affectivity (PAPA). First validation in Italian samples. 16th World Association for Infant Mental Health (WAIMH) Congress (Rome, May 26–30). Infant Mental Health J. 39:311.

[B12] Biehle S. N. Mickelson K. D. (2012). First-time parents' expectations about the division of childcare and play. J. Family Psychol. 26, 36–45. 10.1037/a0026608 22182336

[B13] Bruno A. Celebre L. Mento C. Rizzo A. Silvestri M. C. De Stefano R.. (2020). When fathers begin to falter: a comprehensive review on paternal perinatal depression. Int. J. Environ. Res. Public Health 17:1139. 10.3390/ijerph17041139 32053929 PMC7068539

[B14] Cameron E. E. Sedov I. D. Tomfohr-Madsen L. M. (2016). Prevalence of paternal depression in pregnancy and the postpartum: an updated meta-analysis. J. Affect. Disord. 206, 189–203. 10.1016/j.jad.2016.07.044 27475890

[B15] Carlberg M. Edhborg M. Lindberg L. (2018). Paternal perinatal depression assessed by the Edinburgh Postnatal Depression Scale and the Gotland Male Depression Scale: prevalence and possible risk factors. Am. J. Men's Health 12, 720–729. 10.1177/1557988317749071 29350097 PMC6131440

[B16] Chen Y. H. Huang J. P. Au H. K. Chen Y. H. (2019). High risk of depression, anxiety, and poor quality of life among experienced fathers, but not mothers: a prospective longitudinal study. J. Affect. Disord. 242, 39–47. 10.1016/j.jad.2018.08.042 30170237

[B17] Cox J. L. Holden J. M. Sagovsky R. (1987). Detection of postnatal depression: development of the 10-item Edinburgh Postnatal Depression Scale. Br. J. Psychiatry 150, 782–786. 10.1192/bjp.150.6.782 3651732

[B18] Crespi I. Ruspini E. (2015). Transition to fatherhood: new perspectives in the global context of changing men's identities. Int. Rev. Sociol. 25, 353–358. 10.1080/03906701.2015.1078529

[B19] Crittenden P. M. (1979–2007). CARE-Index: Coding Manual. Family Relations Institute. Miami, FL.

[B20] Danielsson U. Johansson E. E. (2005). Beyond weeping and crying: a gender analysis of expressions of depression. Scand. J. Prim. Health Care 23, 171–177. 10.1080/02813430510031315 16162470

[B21] Edmondson O. J. Psychogiou L. Vlachos H. Netsi E. Ramchandani P. G. (2010). Depression in fathers in the postnatal period: assessment of the Edinburgh Postnatal Depression Scale as a screening measure. J. Affect. Disord. 125, 365–368. 10.1016/j.jad.2010.01.069 20163873 PMC2927780

[B22] Field T. (2018). Paternal prenatal, perinatal and postpartum depression: a narrative review. J. Anxiety Depress. 1:102 10.46527/2582-3264.102

[B23] Figueiredo B. Conde A. (2011). Anxiety and depression in women and men from early pregnancy to 3-months postpartum. Arch. Women's Mental Health 14, 247–255. 10.1007/s00737-011-0217-3 21479759

[B24] Fletcher R. J. Matthey S. Marley C. G. (2006). Addressing depression and anxiety among new fathers. Med. J. Aust. 185, 461–463. 10.5694/j.1326-5377.2006.tb00650.x 17137442

[B25] Garfield C. F. (2015). Supporting fatherhood before and after it happens. Pediatrics 135, e528–e530. 10.1542/peds.2014-3747 25560441 PMC4306805

[B26] Goodman J. H. (2004). Paternal postpartum depression, its relationship to maternal postpartum depression, and implications for family health. J. Adv. Nurs. 45, 26–35. 10.1046/j.1365-2648.2003.02857.x 14675298

[B27] Gutierrez-Galve L. Stein A. Hanington L. Heron J. Ramchandani P. (2015). Paternal depression in the postnatal period and child development: mediators and moderators. Pediatrics 135, e339–e347. 10.1542/peds.2014-2411 25560437

[B28] Habib C. (2012). Paternal perinatal depression: an overview and suggestions towards an intervention model. J. Family Stud. 18, 4–16. 10.5172/jfs.2012.18.1.4

[B29] Innamorati M. Pompili M. Gonda X. Amore M. Serafini G. Niolu C. Girardi P. (2011). Psychometric properties of the Gotland scale for depression in Italian psychiatric inpatients and its utility in the prediction of suicide risk. J. Affect. Disord. 132, 99–103. 10.1016/j.jad.2011.02.003 21371756

[B30] Lai B. P. Tang A. K. Lee D. T. Yip A. S. Chung T. K. (2010). Detecting postnatal depression in Chinese men: a comparison of three instruments. Psychiatry Res. 180, 80–85. 10.1016/j.psychres.2009.07.015 20493548

[B31] Leach L. S. Poyser C. Cooklin A. R. Giallo R. (2016). Prevalence and course of anxiety disorders (and symptom levels) in men across the perinatal period: a systematic review. J. Affect. Disord. 190, 675–686. 10.1016/j.jad.2015.09.063 26590515

[B32] Loscalzo Y. Giannini M. Contena B. Gori A. Benvenuti P. (2015). The Edinburgh Postnatal Depression Scale for fathers: a contribution to the validation for an Italian sample. Gener. Hosp. Psychiatry 37, 251–256. 10.1016/j.genhosppsych.2015.02.002 25724271

[B33] Madsen S. A. (2019). Men and perinatal depression. Trends Urol. Men's Health 10, 7–9. 10.1002/tre.681

[B34] Madsen S. A. Juhl T. (2007). Paternal depression in the postnatal period assessed with traditional and male depression scales. J. Men's Health Gend. 4, 26–31. 10.1016/j.jmhg.2006.10.017

[B35] Magovcevic M. Addis M. E. (2008). The masculine depression scale: development and psychometric evaluation. Psychol. Men Mascul. 9:117 10.1037/1524-9220.9.3.117

[B36] Martin L. A. Neighbors H. W. Griffith D. M. (2013). The experience of symptoms of depression in men vs women: analysis of the national comorbidity survey replication. JAMA Psychiatry 70, 1100–1106. 10.1001/jamapsychiatry.2013.1985 23986338

[B37] Massoudi P. Hwang C. P. Wickberg B. (2013). How well does the Edinburgh postnatal depression scale identify depression and anxiety in fathers? A validation study in a population based Swedish sample. J. Affect. Disord. 149, 67–74. 10.1016/j.jad.2013.01.005 23499163

[B38] Matthey S. (2008). Using the Edinburgh postnatal depression scale to screen for anxiety disorders. Depress. Anxiety 25, 926–931. 10.1002/da.20415 18041072

[B39] Matthey S. Barnett B. Howie P. Kavanagh D. J. (2003). Diagnosing postpartum depression in mothers and fathers: whatever happened to anxiety? J. Affect. Disord. 74, 139–147. 10.1016/S0165-0327(02)00012-5 12706515

[B40] Matthey S. Barnett B. Kavanagh D. J. Howie P. (2001). Validation of the Edinburgh postnatal depression scale for men, and comparison of item endorsement with their partners. J. Affect. Disord. 64, 175–184. 10.1016/S0165-0327(00)00236-6 11313084

[B41] Musser A. K. Ahmed A. H. Foli K. J. Coddington J. A. (2013). Paternal postpartum depression. What Health Care Providers Should Know. J. Pediatr. Health Care 27, 479–485. 10.1016/j.pedhc.2012.10.001 23182851

[B42] Nishimura A. Ohashi K. (2010). Risk factors of paternal depression in the early postnatal period in Japan. Nurs. Health Sci. 12, 170–176. 10.1111/j.1442-2018.2010.00513.x 20602688

[B43] O'Brien A. P. McNeil K. A. Fletcher R. Conrad A. Wilson A. J. Jones D.. (2017). New fathers' perinatal depression and anxiety—treatment options: an integrative review. Am. J. Men's Health 11, 863–876. 10.1177/1557988316669047 27694550 PMC5675308

[B44] Paulson J. F. Bazemore S. D. (2010). Prenatal and postpartum depression in fathers and its association with maternal depression. JAMA 303, 1961–1969. 10.1001/jama.2010.605 20483973

[B45] Paulson J. F. Bazemore S. D. Goodman J. H. Leiferman J. A. (2016). The course and interrelationship of maternal and paternal perinatal depression. Arch. Women's Ment. Health 19, 655–663. 10.1007/s00737-016-0598-4 26790687 PMC4957140

[B46] Poromaa I. Comasco E. Georgakis M. K. Skalkidou A. (2017). Sex differences in depression during pregnancy and the postpartum period. J. Neurosci. Res. 95, 719–730. 10.1002/jnr.23859 27870443 PMC5129485

[B47] Psouni E. Agebjörn J. Linder H. (2017). Symptoms of depression in Swedish fathers in the postnatal period and development of a screening tool. Scand. J. Psychol. 58, 485–496. 10.1111/sjop.12396 29052228

[B48] Quilici M. (2010). Storia Della Paternità. Dal Pater Familias al Mammo. Roma: Fazi.

[B49] Ramchandani P. Psychogiou L. (2009). Paternal psychiatric disorders and children's psychosocial development. Lancet 374, 646–653. 10.1016/S0140-6736(09)60238-5 19411102

[B50] Ramchandani P. Stein A. Evans J. O'Connor T. G. ALSPAC Study Team. (2005). Paternal depression in the postnatal period and child development: a prospective population study. Lancet 365, 2201–2005. 10.1016/S0140-6736(05)66778-5 15978928

[B51] Ramchandani P. Stein A. O'Connor T. Heron J. Murray L. Evans J. (2008). Depression in men in the postnatal period and later child psychopathology: a population cohort study. J. Am. Acad. Child Adolesc. Psychiatry 47, 390–398. 10.1097/CHI.0b013e31816429c2 18388761 PMC2650418

[B52] Rice S. M. Fallon B. J. Aucote H. M. Möller-Leimkühler A. M. (2013). Development and preliminary validation of the male depression risk scale: furthering the assessment of depression in men. J. Affect. Disord. 151, 950–958. 10.1016/j.jad.2013.08.013 24051100

[B53] Seidler Z. E. Dawes A. J. Rice S. M. Oliffe J. L. Dhillon H. M. (2016). The role of masculinity in men's help-seeking for depression: a systematic review. Clin. Psychol. Rev. 49, 106–118. 10.1016/j.cpr.2016.09.002 27664823

[B54] Sweeney S. MacBeth A. (2016). The effects of paternal depression on child and adolescent outcomes: a systematic review. J. Affect. Disord. 205, 44–59. 10.1016/j.jad.2016.05.073 27414953

[B55] Trethowan W. H. Conlon M. F. (1965). The couvade syndrome. Br. J. Psychiatry 111, 57–66. 10.1192/bjp.111.470.57 14261730

[B56] Walsh T. B. Davis R. N. Garfield C. (2020). A call to action: screening fathers for perinatal depression. Pediatrics 145:e20191193. 10.1542/peds.2019-1193 31879278

[B57] Wee K. Y. Skouteris H. Richardson B. McPhie S. Hill B. (2015). The inter-relationship between depressive, anxiety and stress symptoms in fathers during the antenatal period. J. Reprod. Infant Psychol. 33, 359–373. 10.1080/02646838.2015.1048199

[B58] Wynter K. Rowe H. Fisher J. (2013). Common mental disorders in women and men in the first six months after the birth of their first infant: a community study in Victoria, Australia. J. Affect. Disord. 151, 980–985. 10.1016/j.jad.2013.08.021 24119921

[B59] Zierau F. Bille A. Rutz W. Bech P. (2002). The Gotland male depression scale: a validity study in patients with alcohol use disorder. Nordic J. Psychiatry 56, 265–271. 10.1080/08039480260242750 12470317

